# Editorial: looking for the best treatment of bicuspid aortic valves

**DOI:** 10.1093/icvts/ivac253

**Published:** 2022-10-13

**Authors:** Ruggero De Paulis

**Affiliations:** Dept. of Cardiac Surgery, European Hospital, Unicamillus International Medical University, Rome, Italy

**Keywords:** Aortic valve, Valve repair, Valve replacement, Bicuspid valve

In this issue of the *ICVTS*, Prinzig *et al*. [1] report a comparison of 2 relatively new approaches in the treatment of regurgitant aortic valve. From 1 side they evaluate the early haemodynamic results in bicuspid aortic valve (BAV) that were deemed suitable for such approach. In this case, suitability usually refers to the absence of calcification, absence of tissue retraction and good tissue pliability, generally associated with a relatively good symmetry of the BAV. On the other hand, they evaluated the AVNeo procedure (i.e. aortic valve replacement with autologous pericardium) for those cases unsuitable for repair. Although the total number of cases might appear rather limited or inequal in their respective size (61 vs 22 patients), their results leave space for some consideration and comments.

Early functional and haemodynamic results are certainly important for an initial evaluation; the absence of regurgitation coupled with excellent gradient and larger effective orifice area might indicate that satisfactory mid- and long-term results could later be expected. However, comparing valve repair versus valve replacement can be tricky because many confounding factors might play a role. Normally functioning BAV is intrinsically mildly stenotic by definition and it is certainly not surprising that the repair procedure would exacerbate this aspect. Furthermore, the authors have almost exclusively used the sub-commissural annuloplasty to reduce the dimension of the annulus. This technique, nowadays generally abandoned for its unsatisfactory mid-term results [2], might also increase the risk of annular overreduction especially if performed too deep into the interventricular triangles. Given these considerations, a mean gradient of ∼13 mmHg was to be expected after a BAV repair. On the other hand, AVNeo procedure in patients with bicuspid valve has already demonstrated its feasibility [3] and it comes to no surprise that 3 pericardiac leaflets directly sutured to the crescent shape of the aortic annulus guarantee low postoperative gradients.

Whether the AVNeo procedure can be considered a good option in the treatment of un-reparable bicuspid valve is difficult to say and the data reported by Prinzig *et al.* are of little help in this direction. While properly selected bicuspid valve, when treated following an anatomical repair [4] and/or by an adequate annuloplasty [5] are known to be durable for more than 15 years, it still unknown the potential durability of the AVNeo procedure. Although good medium-term results have been reported by the originator of the AVNeo procedure [6], still more data are certainly needed to understand what type of patients would benefit most from this technique or if it could be a valid alternative not only to valve repair but also to valve replacement. Furthermore, when considering the results in patients were the AVNeo procedure has been done in patients with a bicuspid (or unicuspid) valve configuration, the data are even scarcer.

Innovation often comes at the price of patience, perseverance and continuous analysis and evaluation of the results. As always, time will tell.
